# Different Effects of Sleep Deprivation and Torpor on EEG Slow-Wave Characteristics in Djungarian Hamsters

**DOI:** 10.1093/cercor/bhx020

**Published:** 2017-02-07

**Authors:** V. V. Vyazovskiy, S. Palchykova, P. Achermann, I. Tobler, T. Deboer

**Affiliations:** 1 Department of Physiology, Anatomy and Genetics, University of Oxford, OX1 3PT Oxford, UK; 2 Institute of Pharmacology and Toxicology, University of Zurich, CH-8057 Zurich, Switzerland; 3 Laboratory for Neurophysiology, Department of Molecular Cell Biology, Leiden University Medical Center, LUMC S-05-P, PO Box 9600, 2300 RC Leiden, the Netherlands

**Keywords:** Djungarian hamsters, EEG, sleep, slow waves, torpor

## Abstract

It has been shown previously in Djungarian hamsters that the initial electroencephalography (EEG) slow-wave activity (power in the 0.5–4.0 Hz band; SWA) in non-rapid eye movement (NREM) sleep following an episode of daily torpor is consistently enhanced, similar to the SWA increase after sleep deprivation (SD). However, it is unknown whether the network mechanisms underlying the SWA increase after torpor and SD are similar. EEG slow waves recorded in the neocortex during sleep reflect synchronized transitions between periods of activity and silence among large neuronal populations. We therefore set out to investigate characteristics of individual cortical EEG slow waves recorded during NREM sleep after 4 h SD and during sleep after emergence from an episode of daily torpor in adult male Djungarian hamsters. We found that during the first hour after both SD and torpor, the SWA increase was associated with an increase in slow-wave incidence and amplitude. However, the slopes of single slow waves during NREM sleep were steeper in the first hour after SD but not after torpor, and, in contrast to sleep after SD, the magnitude of change in slopes after torpor was unrelated to the changes in SWA. Furthermore, slow-wave slopes decreased progressively within the first 2 h after SD, while a progressive increase in slow-wave slopes was apparent during the first 2 h after torpor. The data suggest that prolonged waking and torpor have different effects on cortical network activity underlying slow-wave characteristics, while resulting in a similar homeostatic sleep response of SWA. We suggest that sleep plays an important role in network homeostasis after both waking and torpor, consistent with a recovery function for both states.

## Introduction

Sleep is a strictly regulated process. The need for sleep (“sleep pressure”) increases in proportion to the duration of preceding waking, and dissipates during subsequent sleep in proportion to its duration and intensity (Tobler 2005). The best characterized physiological indicator of sleep-wake history in mammals is the level of cortical electroencephalography (EEG) slow-wave activity (SWA, EEG power between 0.5 and 4.0 Hz) during non-rapid eye movement (NREM) sleep (Borbély and Achermann 2011). NREM sleep SWA correlates with sleep pressure, being high in early sleep and after sleep deprivation (SD), and decreasing progressively to low levels in late sleep in many mammals (Tobler and Borbély 1986; Huber et al. 2000; Tobler 2005; Borbély and Achermann 2011). It has therefore been proposed that SWA may reflect restorative processes typically associated with sleep (Reimund 1994; Benington and Heller 1995; Inoue et al. 1995; Mackiewicz et al. 2007; Maret et al. 2007; Mackiewicz et al. 2008; Vyazovskiy and Harris 2013; Sanchez-Vives and Mattia 2014; Tononi and Cirelli 2014).

Torpor is a state of hypothermia, which evolved as an adaptation to harsh environmental conditions such as shortage of food or low ambient temperature, allowing survival even in most adverse conditions (Geiser 2013). Daily torpor in Djungarian hamsters is a strictly regulated process, associated with pronounced reductions in behavioral activity, sensory functions, and metabolism. Torpor is typically entered through NREM sleep and in torpor the hamsters appear to be sleeping. Similar to hibernation, bouts of daily torpor are also followed by a period of sleep (Deboer and Tobler 1994). Interestingly, emergence from torpor as well as from a hibernation bout is associated with a substantial increase in EEG SWA during NREM sleep (Daan et al. 1991; Trachsel et al. 1991; Deboer and Tobler 1994, 1996; Strijkstra and Daan 1997; Cerri et al. 2013), resembling the increase after prolonged waking. While the functional role of deep sleep after torpor is debated, one possibility is that it is important for renormalization of network connectivity. Entry into torpor in ground squirrels is associated with a substantial loss of synapses in several brain regions and altered dendritic morphology (Ruediger et al. 2007; von der Ohe et al. 2007), which are restored within 2 h after emergence from torpor (Popov et al. 1992; von der Ohe et al. 2006).

We expect the structural changes at the neuronal network level after torpor to be associated with characteristic changes in the EEG. Previous studies comparing the effects of torpor or hibernation on subsequent sleep were based on EEG spectral analysis (Daan et al. 1991; Deboer and Tobler 1996; Larkin and Heller 1998). However, as well known, EEG spectral analysis does not allow to assess the contribution of specific characteristics of individual slow waves. They in turn reflect underlying network phenomena, such as neuronal population silent periods or network synchronization (Destexhe et al. 1999; Riedner et al. 2007; Vyazovskiy et al. 2007, 2009, 2013; Nir et al. 2011; David et al. 2013; Lemieux et al. 2014; Crunelli et al. 2015; Sheroziya and Timofeev 2015). In the last 2 decades, our knowledge of the neurophysiologic mechanisms underlying the generation of sleep slow waves has increased considerably (Crunelli and Hughes 2009; Buzsaki et al. 2012; Timofeev 2013). Notably, it was found that during NREM sleep cortical networks alternate between periods of generalized population firing and depolarization and relative silence and hyperpolarization (Steriade et al. 2001; Vyazovskiy et al. 2009). Such ON and OFF periods in a local neuronal network population are highly correlated with EEG slow waves (Vyazovskiy et al. 2009). If the changes in EEG slow waves after torpor reflect similar underlying mechanisms as the changes after SD, we hypothesize that the increase in SWA after torpor consists of a combination of the following: (1) an increase in slow-wave incidence, which presumably reflects cortical bistability; (2) an increase in slow-wave amplitude, which may reflect the duration of neuronal OFF periods; and (3) increased slow-wave slopes, which are thought to reflect increased network synchronization (Vyazovskiy et al. 2011a, 2013). To test this hypothesis, we investigated the morphology of individual EEG slow waves recorded in Djungarian hamsters from the parietal and the frontal cortex during baseline NREM sleep, during sleep after spontaneous emergence from an episode of daily torpor, and sleep after a 4-h SD.

## Results

### SWA Increase After Torpor is Associated with Longer, Larger, and Less Complex EEG Slow Waves

**Figure 1.**
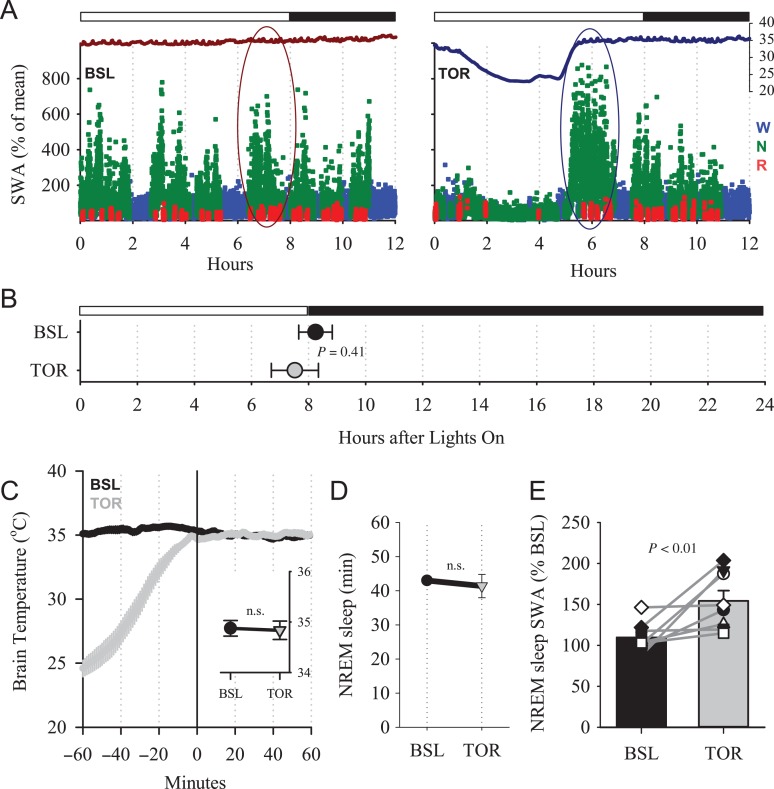
(*A*) Time course of cortical temperature (axis on the right in °C) and parietal EEG SWA (% of 12-h mean value) during the baseline 12-h period without torpor (BSL, left) and during the day with an episode of daily torpor (TOR, right) in an individual hamster. Black and white bar indicates light and dark during the 12-h period. SWA is color coded according to vigilance states (waking: blue, NREM sleep: green, REM sleep: red). The curves at the top are corresponding brain temperature. Note the drop of brain temperature during the episode of daily torpor. Oval areas outline schematically the time interval selected for detailed analyses of slow waves. (*B*) The average time of the interval midpoint selected for the analyses (±SEM). (*C*) Time course of brain temperature during the 1-h interval used for slow-wave analysis (0–60 min) and the preceding hour. Mean values, *n* = 8 hamsters. The inset depicts average brain temperature during NREM sleep. (*D*) Amount of NREM sleep during baseline and after torpor during the time interval used for the analysis of slow waves. (*E*) Effect of torpor on EEG SWA in NREM sleep after emergence from torpor. Mean values + SEM. Gray lines connect values of individual hamsters. The *P* value above: Wilcoxon signed-rank test.

Upon emergence from torpor all animals entered NREM sleep, characterized by increased EEG SWA in the parietal derivation (Fig. 1*A*). For subsequent analyses, we selected for each animal the initial 1-h interval after torpor (Deboer and Tobler 1994), and compared it with the corresponding 1-h interval during baseline. Although it was shown previously (Deboer et al. 2000) that in short photoperiod, the distribution of waking and sleep across 24 h is in general uniform (individual example: Fig. 1*A*), we selected a corresponding time interval in baseline close to the time interval after torpor to minimize potential circadian effects on slow-wave characteristics (Lazar et al. 2015). In most animals, the episode of torpor terminated in the second half of the light period or close to dark onset. Therefore, the corresponding period of baseline sleep that was analyzed occurred around the beginning of the dark period (Fig. 1*B*). Since brain temperature has strong effects on the EEG and cortical network activity (Deboer 1998, 2002; Reig et al. 2010; Sheroziya and Timofeev 2015), we made sure that it was virtually identical during the time intervals of interest, across all vigilance states (Fig. 1*C*), and also specifically within NREM sleep (Fig. 1*C*, inset). The baseline interval we chose was dominated by NREM sleep, and the amount of sleep was not significantly different between the 2 conditions (*P* = 0.62, 2-tailed paired *t*-test). As reported previously (Deboer and Tobler 1994), we confirmed that the initial levels of EEG SWA after torpor were significantly increased, on average by 44.5 ± 15.2% (Fig. 1*D*) compared with baseline, but the magnitude of the SWA increase was variable between individual hamsters (Fig. 1*E*).

**Figure 2.**
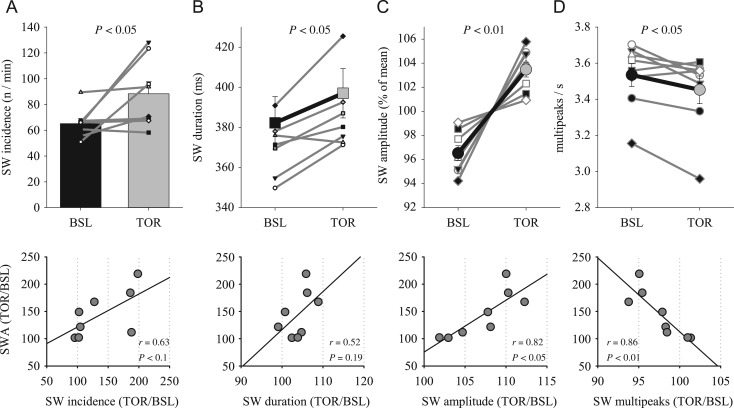
(*A*) The effect of torpor on the incidence of EEG slow waves (SW) in NREM sleep during the first hour after emergence from TOR and the corresponding hour of BSL (as shown in Fig. 1*A*). Mean values + SEM. Thin gray lines connect data from individual hamsters. Bottom: relationship between the effects of torpor on relative slow-wave incidence and corresponding relative SWA. (*B*–*D*) Top: effect of torpor on slow-wave duration, amplitude and the number of peaks during individual slow waves per corresponding slow-wave duration. Bold lines are mean values (*n* = 8, SEM), thin gray lines: individual hamsters. The values of SW amplitude for BSL and TOR conditions are expressed as percentage of mean values between the 2 conditions for each individual animal. Bottom: relationship between relative values of SWA and corresponding slow-wave parameters. Top panels: *P* values correspond to Wilcoxon signed-rank tests. Bottom panels: *r* and *P* values of Pearson's correlation.

It has been shown previously in rats and mice that the time course of EEG SWA across baseline sleep and sleep after SD is associated with characteristic changes in several parameters of individual EEG slow waves (Vyazovskiy et al. 2007, 2011a; Cui et al. 2014). Specifically, the increased levels of absolute SWA were mostly accounted for by a higher incidence of high-amplitude slow waves. Consistently, we found that slow-wave incidence was enhanced in most animals after torpor (Fig. 2*A*, top), although the increase was only weakly correlated with the change in SWA across animals (*P* value of Pearson's correlation = 0.09; Fig. 2*A*, lower panel). Furthermore, slow-wave duration increased moderately in all but 1 individual animal, resulting in a significant increase above baseline (Fig. 2*B*, top panel), but this change did not correlate significantly with the magnitude of the SWA increase (Fig. 2*B*, lower panel). Average slow-wave amplitude increased consistently in all hamsters, and although the change was numerically modest (in most cases not exceeding 10%), it correlated positively with the change in SWA (Fig. 2*C*). Finally, we quantified the occurrence of peaks within individual slow waves. This parameter was shown to reflect the size and complexity of cortical networks simultaneously undergoing slow oscillations (Riedner et al. 2007; Vyazovskiy et al. 2007). Interestingly, the number of peaks within individual slow waves per wave duration decreased significantly after torpor, indicating that the waves became less complex. Notably, this change showed the strongest association with the change in EEG SWA among all slow-wave parameters (Fig. 2*D*). Thus, the analysis shows that several parameters of slow waves are significantly altered during sleep after torpor, and contribute to the overall increase of SWA as measured by power spectral analysis.

### Slow-Wave Slopes Do not Change After Torpor in Djungarian Hamsters

**Figure 3.**
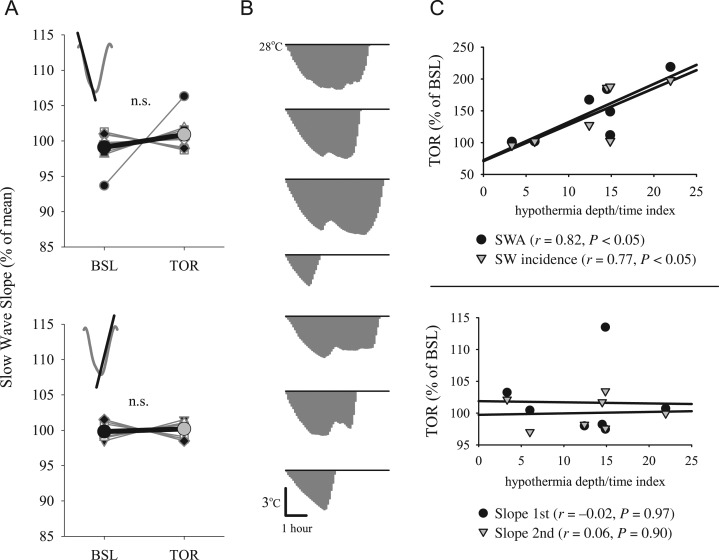
(*A*) The effect of torpor on EEG slow-wave slopes in NREM sleep during the first hour after emergence from TOR and corresponding hour of BSL. Top: first slope, bottom: second slope. Bold lines are mean values (*n* = 8, SEM). Gray thin lines connect data from individual hamsters. The values for BSL and TOR conditions are expressed as percentage of mean values between the 2 conditions. (*B*) Area under the curve used to obtain an index for hypothermia depth/time (expressed as temperature [°C] × time [h]). For each animal, all temperature values below 28°C are integrated to obtain a single value, which was used for correlation analyses shown in *C*. (*C*) Relationship between relative EEG SWA and slow-wave incidence (top) and relative slow-wave slopes (bottom), and the hypothermia depth/time index (°C h). Each symbol represents an individual animal. Straight lines depict linear regression lines. *r* and *P* values: Pearson's correlation.

EEG slow-wave slopes are a sensitive measure reflecting synchrony within local cortical networks (Vyazovskiy et al. 2009). In rats and mice, both the first and the second slope of the EEG slow wave, for example, from its beginning to the maximal peak, and from the maximal peak to the end, are steep during the initial part of the baseline sleep period, as well as after SD, when physiological sleep pressure is increased (Vyazovskiy et al. 2007; Cui et al. 2014). Because slow-wave slopes are not independent of slow-wave amplitude, an amplitude-matching procedure has to be employed to enable comparisons between baseline and the corresponding SD or torpor (Riedner et al. 2007; Vyazovskiy et al. 2007). Unexpectedly, determining slow-wave slopes during sleep after torpor revealed no significant change from baseline, despite substantially increased SWA during the corresponding interval (Fig. 3*A*), and consistently increased slow-wave amplitudes. Although we ensured that average brain temperature was not different between the time interval after torpor and the baseline interval, we further calculated the correlation between temperature and slow-wave slopes after torpor and found no significant association for either the first or the second slope (all *P* values of Pearson's correlation > 0.26).

Next we hypothesized that the duration of the hypothermic state as well as the degree of hypothermia prior to sleep may contribute to the effects we observed. Previously, it was found that the length of torpor episodes is strongly correlated with the increase in SWA during subsequent sleep (Deboer and Tobler 1996). Now we combined the measures of torpor length and the degree of cooling during torpor by integrating the area above the temperature curve below 28 °C, which yielded a single measure of how long the animals had been in hypothermia and how low the temperature was during that time (“hypothermia depth/time”, expressed in temperature [°C] × time [h], Fig. 3*B*). The animals varied remarkably in this respect, and the hypothermia depth/time index was strongly correlated with SWA during subsequent sleep as well as with slow-wave incidence (Fig. 3*C*, top). Interestingly, however, slow-wave slopes were completely unrelated to the hypothermia depth/time index (Fig. 3*C*, bottom).

### Effects of SD on EEG Slow-Wave Characteristics in Djungarian Hamsters

**Figure 4.**
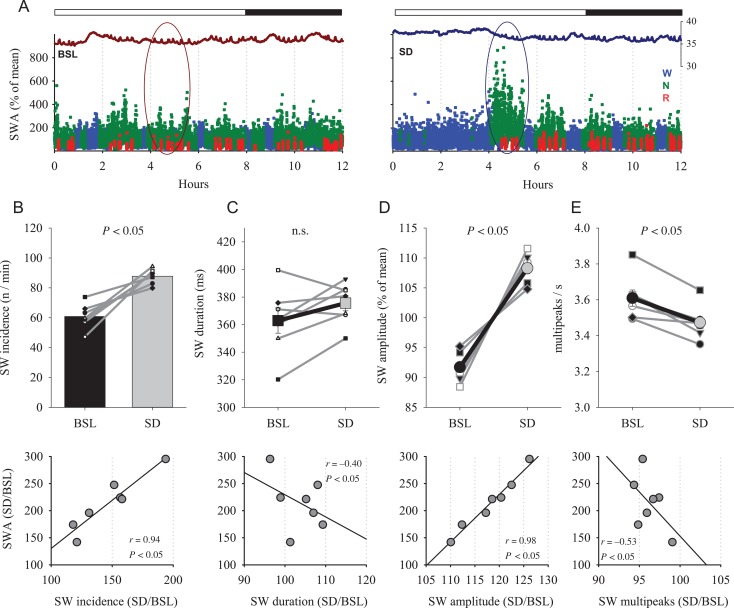
(*A*) Time course of cortical temperature and EEG SWA during BSL and during the 12-h period starting with 4-h SD (right) in an individual hamster. The values of SWA (% of 12-h mean) are color coded according to vigilance states (waking: blue, NREM sleep: green, REM sleep: red). The curves at the top belong to the corresponding brain temperature. (*B*) Top: effect of SD on the incidence of EEG slow waves in NREM sleep during the first hour after SD and corresponding hour of baseline. Mean values, *n* = 7, SEM. Gray lines connect data from individual hamsters. Bottom: the relationship between the effects of SD on relative slow-wave incidence and SWA. (*C*–*E*) Top: effect of SD on the duration of slow waves, their amplitude and the number of peaks within individual slow waves. Bold lines are mean values (*n* = 7, SEM), thin gray lines: individual hamsters. The values of SW amplitude for BSL and SD conditions are expressed as percentage of mean values between the 2 conditions for each individual animal. Bottom: relationship between relative values of SWA and corresponding slow-wave parameters. *P* values correspond to Wilcoxon signed-rank tests (top) and *r* and *P* values of Pearson's correlation (lower panels).

Next we asked whether SD affects slow-wave morphology, and whether the effects are similar to the results after torpor mentioned above. For these analyses, we selected the first 1-h interval after a 4-h SD and the corresponding time interval during the undisturbed baseline recording (individual examples: Fig. 4*A*). As expected and reported previously (Deboer et al. 1994; Palchykova et al. 2002), the initial levels of EEG SWA after SD were significantly increased on average by 114.2 ± 18.8% compared with baseline. The magnitude of SWA increase after SD was significantly higher compared with the increase by 44.5 ± 15.2% (Fig. 1*D*) after torpor (torpor vs. SD: *P* = 0.01, Mann–Whitney *U* test, mixed model ANOVA, interaction “condition × day,” *F*(1,13) = 7.8, *P* = 0.02).

Consistent with previous reports in rats (Vyazovskiy et al. 2007), both incidence of slow waves and their average amplitude increased significantly after SD in the hamsters, by 47.1 ± 10.0% and 18.2 ± 2.1%, respectively (Fig. 4*B,D,* top), with the amplitude increase being significantly higher compared with torpor (*P* = 0.001, Mann–Whitney *U* test, mixed model ANOVA, interaction “condition × day,” *F*(1,13) = 20.55, *P* < 0.01). The duration of single slow waves after SD was not significantly different from baseline (Fig. 4*C*). The magnitude of change did not differ between torpor and SD (*P* = 0.96, Mann–Whitney *U* test). Finally, similar to sleep after torpor, the number of peaks within individual slow waves decreased modestly (Fig. 4*E*, *P* = 0.01), and the change was similar between SD and torpor (*P* = 0.3, Mann–Whitney *U* test). A positive correlation was observed between SWA and both slow-wave incidence and amplitude (Fig. 4*B,D,* lower panel), consistent with data reported previously for the rat (Vyazovskiy et al. 2007). The number of peaks within a wave was unrelated to SWA (Fig. 4*E*, lower panel).

**Figure 5.**
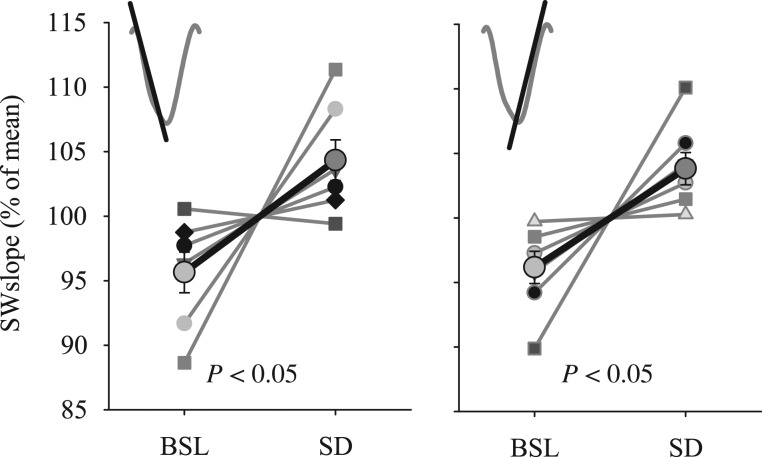
The effect of SD on EEG SW slopes in NREM sleep during the first hour after SD and corresponding hour of BSL. Bold lines are mean values (*n* = 7, SEM). Left: first slope, Right: second slope. Gray thin lines connect data from individual hamsters. The values of SW slopes for BSL and SD conditions are expressed as percentage of mean values between the 2 conditions for each individual animal. *P* values correspond to Wilcoxon signed-rank tests.

Slow waves had steeper slopes after SD compared with baseline in most individual hamsters, resulting in a statistically significant increase (Fig. 5). The magnitude of the change of slopes after SD was 9.4 ± 3.5% for the first slope and 8.2 ± 2.8% for the second slope, which was greater than the corresponding increase after torpor (1.9 ± 1.9% and 0.39 ± 0.9%, *P* = 0.05 and *P* = 0.009, respectively, Mann–Whitney *U* test, mixed model ANOVA, interaction “condition × day,” first slope: *F*(1,13) = 3.9, *P* = 0.07; second slope: *F*(1,13) = 8.4, *P* = 0.01), and was comparable to changes previously reported in rats (Vyazovskiy et al. 2007). We then argued that SD and torpor are not only different behavioral states, but differ also in brain temperature. As is well known, temperature has profound effects on axonal conduction, transmitter release and field potential responses (Moser et al. 1993; Andersen and Moser 1995), which could altogether contribute to changes in the network properties underlying the parameters of slow waves (Sheroziya and Timofeev 2015). To address the potential influence of the difference in brain temperature, we analyzed brain temperature during the first hour after SD and compared it to the corresponding 1-h interval of baseline. Temperature was slightly higher after SD (35.2 ± 0.13°C vs. 34.6 ± 0.1°C, *P* = 0.045), but neither absolute brain temperature nor the difference in temperature between sleep after SD and baseline sleep was associated with the change in slow-wave slopes (all *P* values of Pearson's correlation > 0.3). Thus, we conclude that it is unlikely that the increase in slopes after SD can be accounted for solely by temperature effects.

### Slow-Wave Slopes are Increased After SD and not After Torpor in Two Cortical Areas

**Figure 6.**
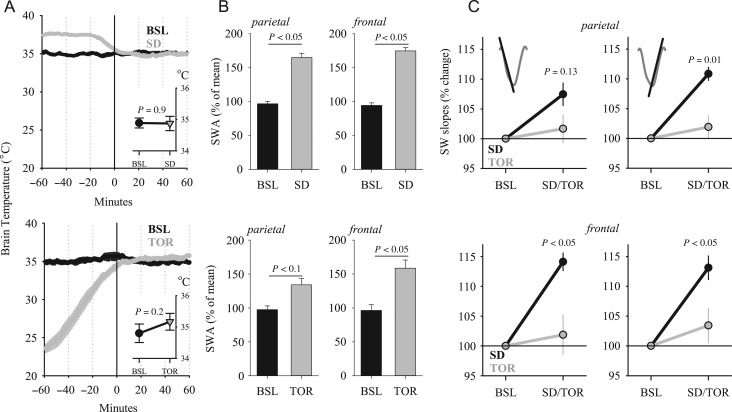
(*A*) Time course of brain temperature during the 1-h interval used for slow-wave analysis (0–60 min) after SD (top) and an episode of daily TOR (bottom) and the preceding hour. Mean values, *n* = 6 hamsters. The inset depicts average brain temperature in NREM sleep during the 1-h interval selected for the analyses. (*B*) The effect of SD (top) and TOR (bottom) on EEG SWA in NREM sleep during the initial hour after SD or TOR and corresponding 1-h BSL interval. SWA values are represented as % of mean value during the corresponding 12-h BSL. *P* values: Wilcoxon signed-rank test. (*C*) The effect of SD and TOR on parietal (top) and frontal (bottom) EEG slow-wave slopes in NREM sleep during the first hour after SD and torpor represented as % of corresponding hour of BSL; *n* = 6, SEM. Left: first slope, Right: second slope. *P* values denote significant differences (Wilcoxon signed-rank test) between the effect of SD and TOR on slow-wave slopes, relative to baseline (= 100%).

The possibility remains that the effects we observe are specific for some cortical areas but not for others. To address this possibility, we analyzed a subset of animals from the data set, in which recordings were performed both after torpor and after SD in the same individual (*n* = 6), and not only in the parietal cortical area, but also in the frontal cortex (Palchykova et al. 2002). Again, we selected the first hour after torpor, and matched this with the corresponding baseline interval by brain temperature (Fig. 6*A*) and the amount of NREM sleep (40.0 ± 3.5 and 35.3 ± 1.9 min). As in the first experiment (Fig. 1*B*), the timing of the interval selected was carefully balanced within each animal (no difference in the timing of the selected interval between baseline and torpor; 7.8 ± 0.9 and 8.0 ± 0.7 h after light onset, n.s.). As expected, SWA was increased both after SD and torpor compared with baseline, in both derivations (Fig. 6*B*; ANOVA for repeated measures: factor “day” *F*(1,5) = 75.24, *P* < 0.001; “condition” *F*(1,5) = 6.6, *P* = 0.05; “day × derivation” *F*(1,5) = 13.4, *P* < 0.05; interaction “condition × day × derivation” n.s.). The increase in SWA after SD was higher compared with torpor in the parietal derivation, but in the frontal EEG SWA values attained a similar level (*P* = 0.03 and 0.44, respectively, Wilcoxon signed-rank test, Fig. 6*B*).

To test within individual animals, whether SD and torpor affect slow-wave characteristics differentially, we investigated the change in the slow-wave amplitude, by including all slow waves in the calculation. The average absolute slow-wave amplitude increased both after SD and torpor when compared with baseline (Supplementary Fig. 1). Next, we calculated average slow-wave slopes, again over all slow waves occurring in NREM sleep during the first 1-h interval after SD and torpor, and during the corresponding baseline interval. This analysis revealed that overall slow-wave slopes increased significantly after SD, while the increase after torpor did not attain statistical significance (first slope: Supplementary Fig. 2, calculations for the second slope yielded similar results).

Since high-amplitude slow waves are most consistently increased after SD, and are reliably associated with neuronal OFF periods (Vyazovskiy et al. 2007, 2009), we next focused on high-amplitude slow waves > 50% (see Methods and Supplementary Fig. 3). We found that the average amplitude of such slow waves was also increased both after SD and torpor (Supplementary Fig. 3). In contrast, the increase of slow-wave slopes, calculated specifically for the slow waves > median amplitude, was mostly observed after SD and attenuated or absent after torpor (first slope: Supplementary Fig. 4, calculations for the second slope yielded similar results).

Since slow-wave slope is not independent of slow-wave amplitude (Supplementary Fig. 5), the changes we observed in the former could merely reflect the changes in the latter, which showed, in general, similar trends (Supplementary Figs. 1–4). However, after the amplitude-matching procedure (see Methods) was employed, we found a significant interaction between factors “day” and “condition” for both slopes (first slope: *F*(1,5) = 13.1, *P* = 0.015; second slope: *F*(1,5) = 11.3, *P* = 0.02). Furthermore, while after SD slow-wave slopes increased significantly in both the parietal and in the frontal derivation (absolute values: Supplementary Fig. 6; relative values: Fig. 6*C*, all *P* values < 0.05, Wilcoxon signed-rank test), in neither derivation a change in slopes was found after torpor (absolute values: Supplementary Fig. 6, relative values: Fig. 6*C*, n.s., Wilcoxon signed-rank test). In addition, the magnitude of the increase in slow-wave slopes was significantly higher after SD when compared with torpor for both the first and the second slope in the frontal derivation, and for the second slope in the parietal EEG (Fig. 6*C*; factor “condition”: first slope *F*(1,5) = 7.8, *P* = 0.038; second slope: *F*(1,5) = 19.0, *P* = 0.007; factor “derivation” or the interaction “condition × derivation” n.s., ANOVA for repeated measures).

To rule out that the difference between SD and torpor could be related to the time of day, we compared slow-wave slopes during baseline between hours 5–6 and 7–8 after light onset, which corresponded to the time intervals used for the analyses after SD or torpor (see Methods). We found no significant change in slopes, equated by amplitude between the intervals (all *P* values > 0.3, Wilcoxon signed-rank test). In addition, since the magnitude of the increase in SWA was significantly lower during sleep after torpor when compared with SD in the parietal derivation, the possibility remains that slow-wave slopes did not change in the former condition because sleep was more “superficial.” To address this possibility, we calculated a correlation between the magnitude of change in SWA and slopes. We posited that if there was an association, those individuals, which showed a pronounced increase in SWA, should also have a greater increase in slopes. However, this was not the case (first slope: *R* = −0.11, *P* = 0.8; second slope: *R* = 0.1, *P* = 0.8), and even in the animal which showed the largest (>2-fold) increase in SWA from baseline to sleep after torpor, the increase in slow-wave slopes was negligible (<1%). In contrast, the increase in SWA after SD correlated positively with the increase in slopes (first slope: *R* = 0.84, *P* < 0.05; second slope: *R* = 0.84, *P* < 0.05).

Interestingly, we found that slow-wave duration was not altered by SD in the parietal derivation, but decreased significantly in the frontal EEG (parietal: 369.3 ± 4.7 ms vs. 362.5 ± 6.2 ms, n.s.; frontal: 393.9 ± 6.0 ms vs. 369.7 ± 5.2 ms, *P* < 0.05). In contrast, after torpor, while slow-wave duration was not affected in the parietal derivation (345.8 ± 5.1 ms vs. 352.1 ± 4.8 ms, n.s.), it was increased in the frontal cortex (379.1 ± 3.0 ms vs. 393.34 ± 3.9 ms, *P* < 0.05). Since in this experiment we recorded the same animals after SD and torpor, we performed the amplitude-matching procedure directly between SD and torpor. Consistently, the duration of slow waves in the frontal derivation was substantially longer after torpor when compared with SD (*P* < 0.01), while both first and second slopes were decreased significantly (*P* < 0.05 and 0.01, respectively).

**Figure 7.**
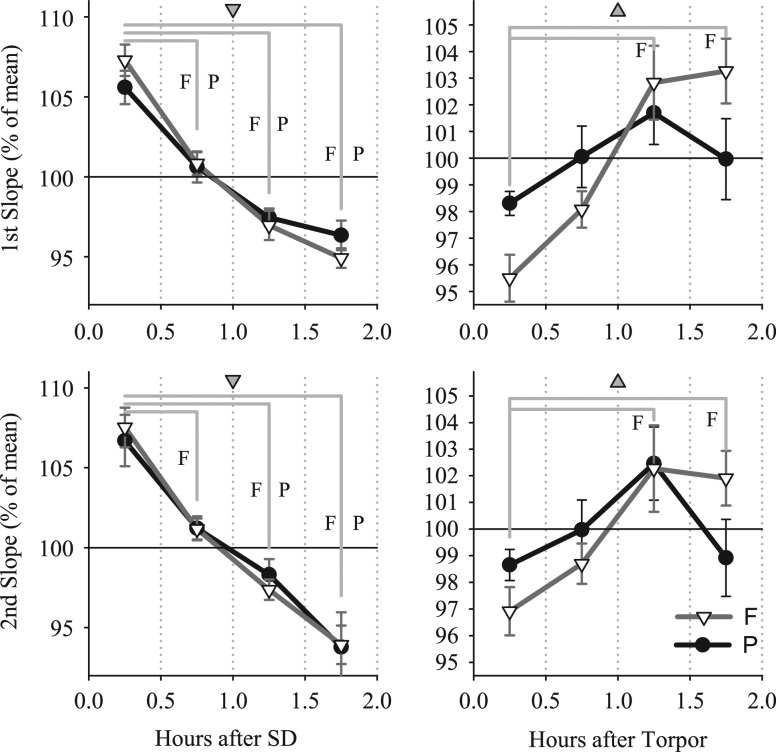
Time course of EEG slow-wave slopes in NREM sleep during the first 2 h after SD (left panels) and after TOR (right panels). Mean values (*n* = 6) ± SEM are shown for 30-min intervals as % of corresponding mean values over the entire 2-h interval. The change in slopes from the first 30-min interval is assessed by post hoc Dunn-Sidak multiple comparison test after significance in one-way ANOVA (gray triangles: *P* < 0.05, the orientation of triangles denotes the directionality of change from the first 30-min interval). For each comparison shown with straight gray lines, the letter F or P corresponds to a significant difference found for the frontal and parietal derivation, respectively.

Since wakefulness and torpor likely lead to distinct changes in synaptic connectivity, differential effects on slow-wave slopes within the initial period of sleep after SD and torpor can be anticipated. To address this possibility, we determined the time course of slow-wave slopes during the first 2 h after SD and emergence from torpor. As expected, after SD a significant decrease in slopes was apparent in both the frontal and the parietal derivation in the course of these 2 h. This was the case for both the first and the second slope (Dunn-Sidak test after significance in one-way ANOVA, factor ‘30-min interval’; Fig. 7). In contrast, no change was apparent in the parietal derivation in the first 2 h after torpor, while slopes in the frontal derivation became progressively steeper (ANOVA for repeated measures, interaction “condition × interval”: parietal: first slope *F*(3,12 = 5.3), *P* = 0.02; second slope *F*(3,12 = 5.7), *P* = 0.01; frontal: first slope *F*(3,12 = 38.4), *P* < 0.001, second slope *F*(3,23 = 20.3), *P* < 0.001). Notably, brain temperature was stable during the initial 2-h interval both after SD and torpor (one-way ANOVA, factor ‘30-min interval,’ n.s.), which rules out the possibility that the changes observed merely mirror the process of warming up.

## Discussion

The aim of this study was to investigate the changes in EEG slow-wave characteristics during NREM sleep after torpor in Djungarian hamsters and to compare these changes with baseline sleep and sleep following SD. Previous studies in this species, as well as in hibernating animals revealed a consistent, substantial increase in EEG SWA during initial sleep after emergence from torpor (Daan et al. 1991; Trachsel et al. 1991; Deboer and Tobler 1994, 2003; Strijkstra and Daan 1997). Neither the mechanisms underlying this increase, nor their functional significance are understood.

As has been found recently, the changes in EEG SWA during spontaneous sleep and during sleep after SD are accounted for by changes in several characteristics of slow waves. Specifically, in early sleep, under increased sleep pressure after a normal waking episode as well as after prolonged waking, slow waves have higher amplitudes, they occur more frequently, and have steeper slopes (Riedner et al. 2007; Vyazovskiy et al. 2007). Important insights have been obtained in follow-up studies in the rat, where a link was established between EEG changes during sleep and the underlying cortical neuronal activity pattern (Vyazovskiy et al. 2011b). Specifically, the data suggest that the first segment of an EEG slow wave (from its beginning to its maximal peak) corresponds to a transition of the network from the period of elevated activity to generalized silence, whereas the second segment (from the maximal peak to slow-wave end) corresponds to the transition from relative population silence to elevated network activity (Steriade et al. 1993; Achermann and Borbely 1997; Amzica and Steriade 1998; Steriade and Amzica 1998; Destexhe et al. 1999; Molle et al. 2002; Vyazovskiy et al. 2009). Notably, the magnitude of the sleep-dependent change differs between specific slow-wave parameters, and their relative contribution to the overall signal accounts well for the observed changes in the EEG power spectra (Riedner et al. 2007; Vyazovskiy et al. 2007).

Here, we investigated for the first time the morphology of NREM sleep slow waves in the Djungarian hamster. We found that slow-wave incidence during sleep after torpor is increased and is strongly associated with the duration and depth of hypothermia (Fig. 1*A* and 3*C*). One possible interpretation of this result is that during sleep after torpor, neuronal populations tend to transition into silent mode more frequently. This may be due to an overall decrease in network activity, which is required to sustain population up-states (Sanchez-Vives and McCormick 2000; Haider et al. 2006). It has been shown previously in rats that the magnitude of change in slow-wave duration as a function of sleep pressure is low compared with the contribution of slow-wave incidence (Vyazovskiy et al. 2007). This finding suggested that during sleep after SD, in addition to the tendency of the network to enter OFF periods, another, opposing process takes place, which forces the network to resume activity, which normally prevents sustained periods of neuronal silence (Vyazovskiy et al. 2013). An essential feature of this process, is the speed and synchronicity of the network transitions from silence to activity, which may arise from local intracortical connectivity, or involve a stronger input from subcortical areas, such as from the thalamus (Crunelli and Hughes 2009; David et al. 2013). Previous studies in rats and mice revealed that slow-wave slopes are steeper after prolonged spontaneous waking or SD, which suggests increased cortical excitability or stronger functional or anatomical connectivity within cortical networks (Vyazovskiy et al. 2009; Cui et al. 2014). Notably, our present study shows that slow-wave slopes are steeper during sleep after SD also in Djungarian hamsters.

Since changes in specific characteristics of cortical EEG slow waves during sleep reflect underlying changes in neuronal activity (Vyazovskiy et al. 2009), sleep after torpor represents a unique model to investigate the network mechanisms—both functional and structural—underlying the homeostatic response of SWA. It has been proposed that prolonged wakefulness is associated with a net increase in synaptic strength, which is manifested in high SWA, arising from increased synchrony among cortical networks (Vyazovskiy and Harris 2013; Tononi and Cirelli 2014). The observation of increased SWA during NREM sleep after torpor (Deboer and Tobler 1994, 2003) appears to contradict this notion, as hibernation or hypothermia are typically associated with a substantial temperature-dependent loss of synapses (Popov et al. 1992; von der Ohe et al. 2006, 2007). It is unknown whether similar morphological changes occur after an episode of daily torpor in Djungarian hamsters, but our results suggest that some aspects of the cortical network activity during sleep after torpor may differ from sleep after SD. Specifically, in this study we observed that slow-wave slopes are not increased during the first hour after an episode of daily torpor, despite substantially elevated SWA during this period, and no significant correlation was observed between the magnitude of the increase in SWA and the change in slope after torpor. Moreover, we observed that the change in slow-wave slopes during the first 2 h of sleep after SD and torpor showed opposite trends, especially in the frontal derivation. Specifically, slow-wave slopes decreased in the course of recovery sleep after SD, while an increase was observed after torpor (Fig. 7). Previous studies in ground squirrels showed that temperature-dependent loss of synaptic connectivity was restored during the first 2 h after return to euthermia (Popov et al. 1992; von der Ohe et al. 2006). Although upon emergence from a bout of hibernation the animals spend most of the time sleeping (Strijkstra and Daan 1997), the role of sleep in the process of renormalization of synaptic connectivity during this time remains to be determined. Interestingly, while the increase in SWA was consistently lower after torpor when compared with SD in the parietal derivation, this was not the case in the frontal EEG, where the levels of SWA did not differ significantly between the 2 conditions. In contrast to the regional difference in SWA, slow-wave slopes did not change after torpor in either derivation (Fig. 6*C*). Furthermore, in contrast to SD, we did not observe an association between SWA and slopes after torpor, suggesting that the lack of change in the latter variable could not be accounted for by less “intense” sleep.

An important aspect that merits further investigation is the relationship between specific characteristics of slow waves recorded in a local cortical region, such as their incidence, amplitude, slopes and duration, and their counterparts at the network level, such as the occurrence of population OFF periods or neuronal synchrony. It has been shown in rats that the amplitude of slow waves is largely determined by the duration of generalized network silence, although, interestingly, after SD the increase in the incidence of high-amplitude slow waves was substantially greater than the change in OFF period duration (Vyazovskiy et al. 2007, 2009, 2013). On the other hand, both the amplitude of slow waves and the duration of OFF periods appear to be determined by the degree of neuronal synchrony, which correlates closely with slow-wave slopes, and increases consistently under high sleep pressure (Vyazovskiy et al. 2009). Previous studies have shown that slow-wave slopes reflect local synchronization among cortical neurons at ON–OFF and OFF–ON transitions (Vyazovskiy et al. 2009). Therefore, the most parsimonious interpretation of our results is that torpor and SD in Djungarian hamsters are associated with changes in local network connectivity. It has also been shown that hibernation is associated with brain-wide morphological changes (von der Ohe et al. 2007), suggesting that large-scale network connectivity may also be affected in this condition. However, as the EEG is not an ideal tool for investigating long-range synchronization, due to possible confounds of volume conduction and a common reference, future experiments with neuronal activity and local field potentials recordings are necessary to provide further insights with respect to local and global network activities after torpor. Furthermore, it is also possible that other factors may have a unique contribution into specific aspects of network activity, affecting the characteristics of slow waves, such as network excitability, inhibition or thalamic inputs (Sanchez-Vives et al. 2010; Chen et al. 2012; David et al. 2013; Lemieux et al. 2014; Crunelli et al. 2015), which all may vary as a function of preceding sleep-wake history or be influenced by hypothermia.

An acknowledged caveat of our study is that slow-wave slopes are not independent of the amplitude (Supplementary Fig. 5), and, therefore, the stronger increase in the former variable may be viewed merely as a consequence of the change in the latter. While this scenario is difficult to rule out entirely, our data suggest that changes in slow-wave slopes can, at least in part, be dissociated from changes in amplitude. Importantly, this was the case for sleep after SD only, when slow-wave slopes were found to increase as compared with baseline, even after the amplitude-matching procedure was employed. In contrast, no change in slopes was apparent after torpor, suggesting that in this case the increase in slopes merely reflected an increased incidence of high-amplitude slow waves. We should point out, however, that from the perspective of the underlying physiology, we find it more compelling that the slope (or, more precisely, the change in the underlying network activity) would predict the amplitude of the corresponding slow wave, rather than the other way around.

It is well known that slopes of evoked potentials are affected by brain temperature (Moser et al. 1993), and brain temperature also profoundly affects the overall network activity as well as frequency of specific EEG/LFP oscillations (Deboer 1998, 2002; Reig et al. 2010; Sheroziya and Timofeev 2015). However, for several reasons, it is unlikely that the effects we report can be due to changes in brain temperature. First, the time intervals selected for the analyses were carefully matched for brain temperature. Second, no significant correlation between slow-wave slopes and brain temperature was found. Finally, if brain temperature was a major determinant of the changes we observed, then it is expected to have similar effects across cortical regions, which was not the case. In contrast, the differences between parietal and frontal derivations indicate that the effects we observed are related to local cortical activities, rather than being unspecific, generalized effects.

The strong association between the time spent in hypothermia and SWA or slow-wave incidence after torpor suggests that the SWA increase during sleep after torpor may reflect a compensatory homeostatic response. This is consistent with the homeostatic regulation of slow waves after torpor that was demonstrated in a selective slow-wave deprivation experiment in Djungarian hamsters (Palchykova et al. 2002). Our present analyses suggest that while network mechanisms underlying the SWA increase after prolonged waking and torpor are in part, different, the consequences of SD and torpor could share an important similarity. Both extended wakefulness and torpor may be associated with changes in the architecture and dynamics of cortical circuitry, which require renormalization. Sleep may provide the tools and physiological environment for this renormalization process, consistent with the suggested recovery function of sleep. In this case, sleep with high-amplitude slow waves may represent a common endpoint for qualitatively very different states, emphasizing its fundamentally important role for network homeostasis.

## Methods

### Animals

Three experimental groups of adult male Djungarian hamsters (*Phodopus sungorus*) were used (*n* = 8 baseline and torpor; *n* = 7 baseline and SD; and *n* = 6 baseline, torpor, and SD, respectively). Chronic EEG and electromyogram (EMG) recordings were performed in freely behaving unrestrained hamsters kept individually in Macrolon cages (36 × 20 × 35 cm) with food and water available ad libitum, and maintained in an 8 h light–16 h dark cycle [light from 09:00 to 17:00 h; 7 W OSRAM DULUX EL energy saving lamp (Osram, Germany), ~30 lux]. Mean ambient temperature was between 14 and 18°C. Rest-activity behavior of all animals was monitored continuously with IR-sensors (Deboer et al. 1994; Deboer and Tobler 2003) and weight and pelage color were scored every week (Figala et al. 1973). After 10 weeks, those animals showing pronounced adaptation to the short photoperiod based on rest-activity behavior, decreased body weight, and an increase in fur color index were implanted i.p. with temperature sensitive transmitters to continuously record body temperature (Mini Mitter). Hamsters exhibiting regular torpor bouts evident in their body temperature records were selected for implantation of EEG and EMG recording electrodes and thermistors to record brain temperature.

### Surgical Procedure

Under deep ketamine-xylasine anesthesia, the animals were implanted with gold-plated miniature screws (0.9 mm diameter) inserted into the skull that served as EEG electrodes. Screws were placed epidurally over the right parietal cortex (data sets 1–3:2 mm lateral to midline and 2 mm posterior to bregma) and the right frontal cortex (data set 3:2 mm lateral to midline and 2 mm anterior to bregma). In all animals, a reference electrode was placed over the cerebellum (2 mm posterior to lambda, on midline). A calibrated thermistor (Thermometrics, Inc., P20, R 25°C at 1 kW, maximum diameter 0.5 mm, accuracy ± 0.05°C) was inserted between the skull and dura through a hole over the left frontal cortex (2–3 mm lateral to midline and 2 mm anterior to bregma) to record cortical brain temperature. Two gold wires inserted into the neck muscles (diameter 0.2 mm) served to record the EMG. The electrodes and thermistor were connected to stainless steel wires that were fixed to the skull with dental cement (Deboer et al. 1994). At least 1 week was allowed for recovery before the recording was commenced.

### Signal Processing and Analysis

EEG and EMG acquisition was continuous, while analysis, and scoring of the 3 vigilance states, NREM sleep, REM sleep, and waking, was based on 4-s epochs as previously described (Deboer and Tobler 1994; Palchykova et al. 2002). The EEG and the EMG signals were amplified (amplification factor ~2000), conditioned by analog filters (high-pass filter: −3 dB at 0.016 Hz; low-pass filter: −3 dB at 40 Hz, less than −35 dB at 128 Hz) sampled either with 256 Hz (EEG digitally low-pass filtered at 25 Hz) or with 512 Hz (digitally filtered, EEG: low-pass FIR filter 25 Hz; EMG: band-pass FIR filter 20–50 Hz) and stored with a resolution of 128 Hz. EEG records for consecutive 4-s epochs were subjected to a Fast Fourier Transform routine to obtain EEG power spectra (0.25-Hz bins resolution). Sleep stages were scored visually based on the EEG and EMG signals. Waking was characterized by a low voltage, high-frequency EEG pattern, and phasic EMG activity. NREM sleep was characterized by the occurrence of high-amplitude slow waves and low tonic EMG activity. During REM sleep, the EEG was similar to that during waking, but only heart beats and occasional twitches were evident in the EMG signal. Epochs containing EEG artifacts recognized during visual scoring were excluded from signal analysis: data set 1: baseline 2.8 ± 0.5%, torpor 2.5 ± 0.5% of total recording time (TRT), of which 84.4 ± 8.4% and 84.9 ± 8.2%, respectively, occurred during waking (W); data set 2: baseline 2.6 ± 0.6%, SD 2.4 ± 0.4% of TRT (W: 59.8 ± 6.7% and 85.6 ± 4.2% respectively); data set 3: torpor experiment, baseline 11.7 ± 2.5% (W: 81.6 ± 3.4%), torpor day 7.92 ± 1.4% of TRT (W: 41.2 ± 7.0 %); SD experiment: baseline 14.6 ± 1.4% of TRT (W: 68.1 ± 2.5%); SD day 21.4 ± 1.5% of TRT (W: 83.5 ± 1.5%).

### Experimental Design

Most of the analyses were performed on NREM sleep during the 1-h interval immediately following torpor or SD. In the first data set, the animals spent 4.8 ± 0.3 h in torpor, defined as <32°C (Ruf et al. 1989; Deboer and Tobler 1994), where the average temperature during torpor was 26.1 ± 0.5°C, and the minimum reached was 23.0 ± 0.33°C. In the third data set, the animals spent on average 7.3 ± 0.8 h in torpor, where the average temperature during the torpor bout was 22.3 ± 0.5°C, and the average minimum reached was 19.6 ± 0.6°C. As it cannot be excluded that brain temperature or the time of day can influence specific slow-wave characteristics, special care was taken to ensure that brain temperature was not different between the conditions, and the time interval selected during baseline was matched as closely as possible with respect to its timing after light onset and the amount of NREM sleep, which contributed to the analyses. In the third data set, a significant variability in torpor duration was observed. In 3 animals, the “recovery” interval occurred in the light period, and in the remaining 3 animals during the dark period. The baseline interval was selected, correspondingly, during the light and during the dark period, and the amount of data contributing to the mean between all animals collected during the light and dark period was precisely matched. Paired *t*-tests within each subgroup revealed that slow-wave slopes were not significantly affected by torpor in either case (*P* > 0.2), and the change in slopes after torpor relative to baseline was not different between the light and the dark phase (all *P* values > 0.2). Due to the low number of animals contributing to each subgroup (light and dark, *n* = 3 and 3, respectively), these results should be interpreted with caution.

SD was attained by tapping on the cage and by introducing objects (e.g., nesting material) into the cage whenever the animal appeared drowsy or assumed a sleeping position. SD was successful in both data sets 2 and 3 (98.9 ± 0.24% and 99.9 ± 0.1% time was spent in waking, respectively).

### Slow-Wave Analysis

In order to investigate the occurrence and characteristics of EEG slow waves, individual waves were detected and analyzed during baseline NREM sleep, during initial sleep after emergence from torpor and after SD. Detection of individual waves was performed on the EEG signal after band-pass filtering (0.5–4 Hz, stopband edge frequencies 0.1–8 Hz) with MATLAB filtfilt function exploiting a Chebyshev Type II filter design (MATLAB, The Math Works, Inc.) (Achermann and Borbély 1997; Vyazovskiy et al. 2007, 2009). Filtering occurred in forward and reverse direction resulting in zero phase shift of the filtered signal. Slow waves were detected as negative deflections of the filtered EEG signal between 2 consecutive positive deflections above zero crossing. Some of the analyses were performed on all slow waves during the time interval of interest (Supplementary Figs. 1, 2), and most of the calculations were performed on those waves with above median amplitude, defined as the most negative value within each slow wave, across all conditions (e.g., baseline, torpor, SD). We selected the top 50% of slow waves for the final analyses, because previous studies showed that low-amplitude slow waves do not show consistent trends across the sleep period or after SD, and are at most weakly related to OFF periods in neuronal activity (Vyazovskiy et al. 2007, 2009). However, the effects of SD and torpor on slow-wave slopes were consistent, irrespective of whether all slow waves were included in the analyses (Supplementary Fig. 2), or only those above median amplitude (Supplementary Fig. 4). Furthermore, plotting the distributions of slow-wave slopes for all slow waves or slow waves above median amplitude did not reveal obvious differences which could have biased our analyses.

Slow-wave slopes were calculated for the first and second segment of the wave, defined as the part of the wave from its start to its most negative peak, and from the most negative peak to the end of the slow wave, respectively. Since the amplitude and the slope of slow waves are not independent (Supplementary Fig. 5), in addition to the median amplitude threshold, an amplitude-matching procedure was employed, as previously (Vyazovskiy et al. 2007). This procedure consisted of an iterative process whereby each individual slow wave in one of the conditions was “paired” with its closest amplitude match found in the second condition. Subsequently, corresponding average slow-wave slopes were compared. As a result of this procedure, the average amplitude of slow waves included in the analyses was virtually identical between the conditions. At the same time, the observed differences, or lack thereof, in slopes were not directly confounded by a difference between amplitudes because, by definition, all amplitudes found in the distribution contributed equally to the analyses. All the analyses and comparisons between conditions were performed independently for each of the 2 derivations (frontal and parietal). Since EEG signals are affected by volume conduction and a common reference, which may lead to spurious correlations between signals recorded from 2 distant sites, our electrode montage prevented us from obtaining reliable estimates of the effects of torpor or SD on long-range network synchronization.

### Statistics

Statistical analyses were performed in MATLAB (The Math Works, Inc.) or SPSS 22.0 (IBM). All values reported are mean ± SEM. Unless stated otherwise, for within-subject comparisons, one-, two-, or three-way ANOVAs for repeated measures were used (with factors “condition [SD experiment, torpor experiment],” “day [baseline, SD/torpor],” “time interval,” and/or “derivation [frontal, parietal]”). For the analyses shown in Figure 7, 1 individual hamster was excluded from calculating ANOVA, because it did not sleep during the second 30-min interval after torpor. Visual inspection confirmed that the remaining values in this animal were in the range of other individual hamsters, and filling the missing value with an interpolated value (calculated by averaging the preceding and the following value) did not alter numerical or statistical results noticeably. For between-subject comparisons, mixed design ANOVA was used, with between-subject factor “condition (SD, torpor)” and within-subject factor “day” (baseline, SD/torpor).

## Supplementary Material

Supplementary Data
