# Supplementary material for: Different Effects of Sleep Deprivation and Torpor on EEG Slow-Wave Characteristics in Djungarian Hamsters
Source: Cereb Cortex. 2017 Feb 7;27(2):950–61. doi: 10.1093/cercor/bhx020 (PMC5390404; doi:10.1093/cercor/bhx020)
Supplement: Supplementary Data [file CerCor-2016-00471R2SupplFigures.pdf]

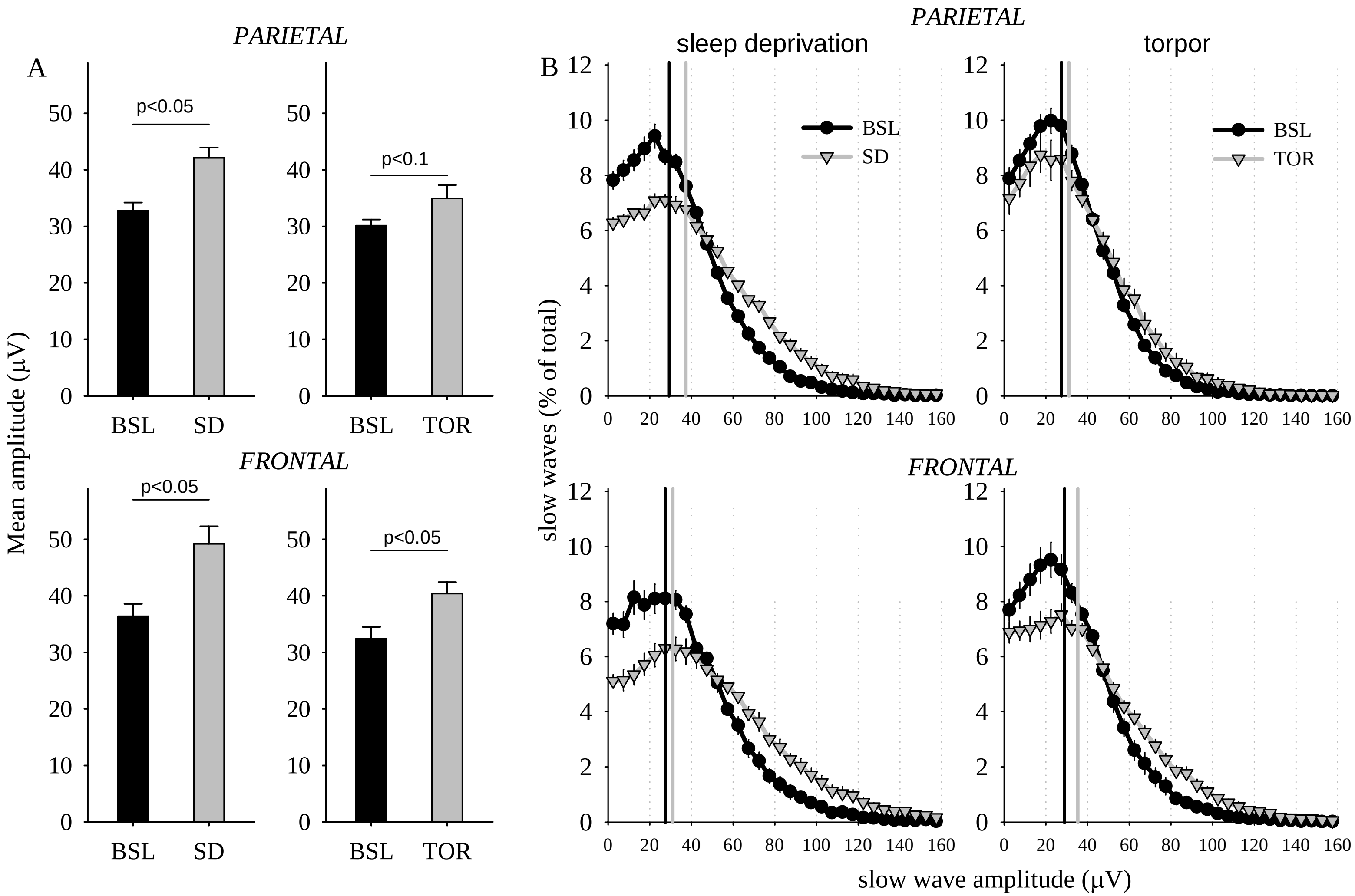

Suppl. Figure 1 (A) Effect of sleep deprivation (SD) and torpor (TOR) on the absolute amplitude of NREM sleep slow waves. Mean values (SEM, n=6) of the amplitude of all slow waves occurring during the first 1-h interval after SD or TOR and corresponding baseline interval (BSL). Total number of slow waves contributing to these analyses was as follows. SD experiment: parietal, BSL 5698.8±394.22, SD 6206.3±605.45; frontal: BSL 5438.2±414.48; SD 6190.3±624.13; TOR experiment: parietal, BSL 6246±380.66, TOR 6548±690.07; frontal: BSL 5796.8±344.74; TOR 5858.7±552.31. P-values above the curves correspond to Wilcoxon signed-rank test (ANOVA for repeated measures: parietal, 'condition' F(1.5)=39.1, p<0.01; 'day' F(1,5)=5.5, p=0.07, 'condition*day' F(1,5)=5.7, p=0.06; frontal: 'condition' F(1.5)=64.7, p<0.001; 'day' F(1,5)=6.3, p=0.053, 'condition*day' n.s.). (B) Distribution of all NREM sleep slow waves during the first 1-h interval after SD (left panels) and TOR (right panels), and the corresponding BSL interval, as a function of amplitude. Mean values (SEM, n=6) are shown for the parietal (top panels) and the frontal derivation (bottom panels). Vertical lines correspond to the average median amplitude (50% threshold, n=6; black: baseline, grey: SD or TOR).

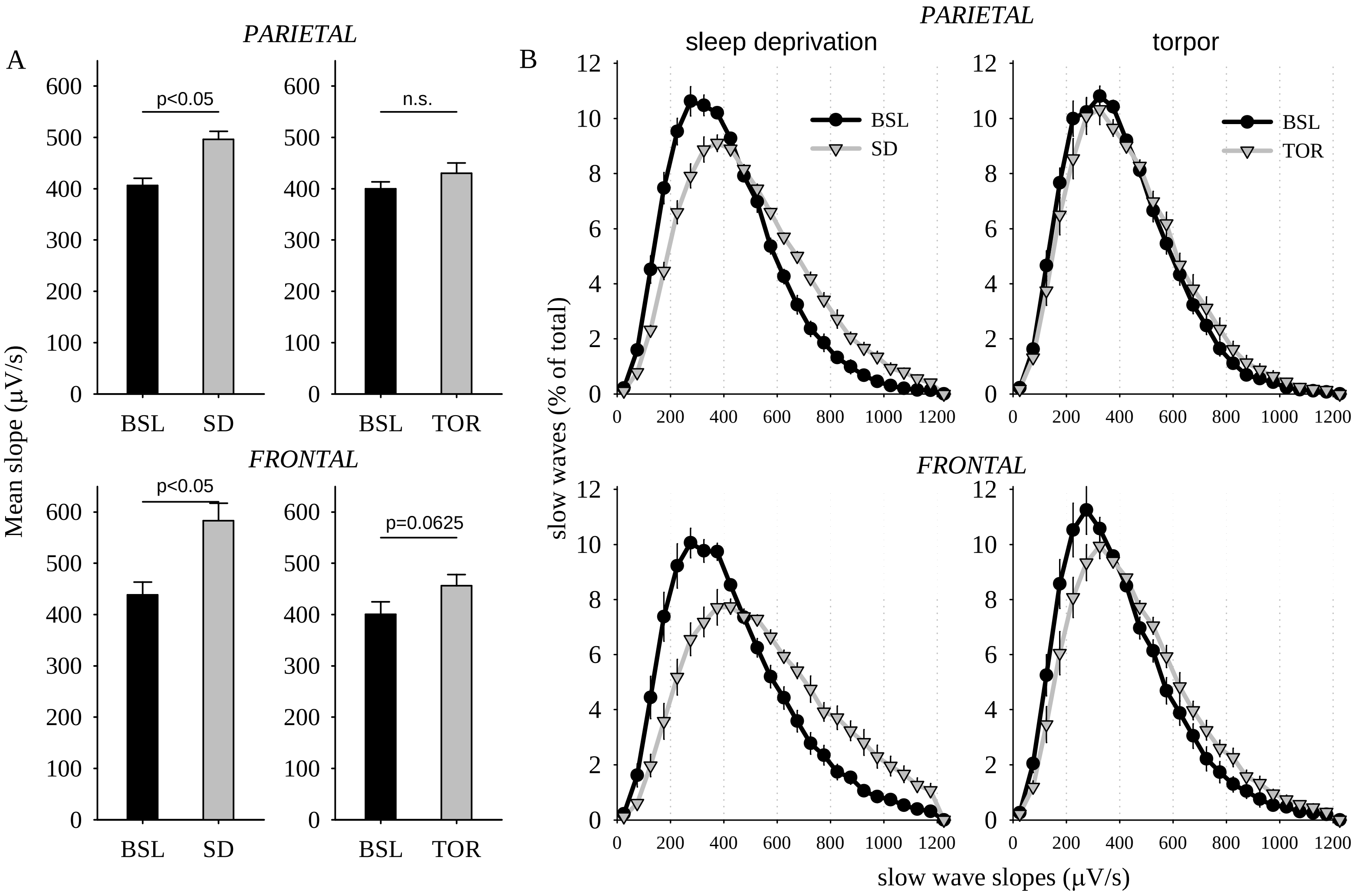

Suppl. Figure 2 (A) Effect of sleep deprivation (SD) and torpor (TOR) on the 1st slope of NREM sleep slow waves. Mean values (SEM, n=6) of the slope of all slow waves occurring during the first 1-h interval after SD or TOR. P-values above the curves correspond to Wilcoxon signed-rank test (repeated measures ANOVA: parietal, 'day' $F(1,5)=55.6$, $p<0.01$; 'condition' (SD,TOR)': $F(1,5)=4.1$, $p=0.1$; 'day*condition' $F(1,5)=9.9$, $p=0.025$; frontal: 'day' $F(1,5)=46.6$, $p<0.01$; 'condition' $F(1,5)=10.0$, $p=0.03$; 'day*condition' $F(1,5)=8.0$, $p=0.04$). (B) Distribution of all NREM sleep slow waves during the first 1-h interval after SD (left panels) and after TOR (right panels), and the corresponding baseline (BSL) interval, as a function of slope. Mean values (SEM, n=6) are shown for the parietal (top panels) and the frontal derivation (bottom panels).

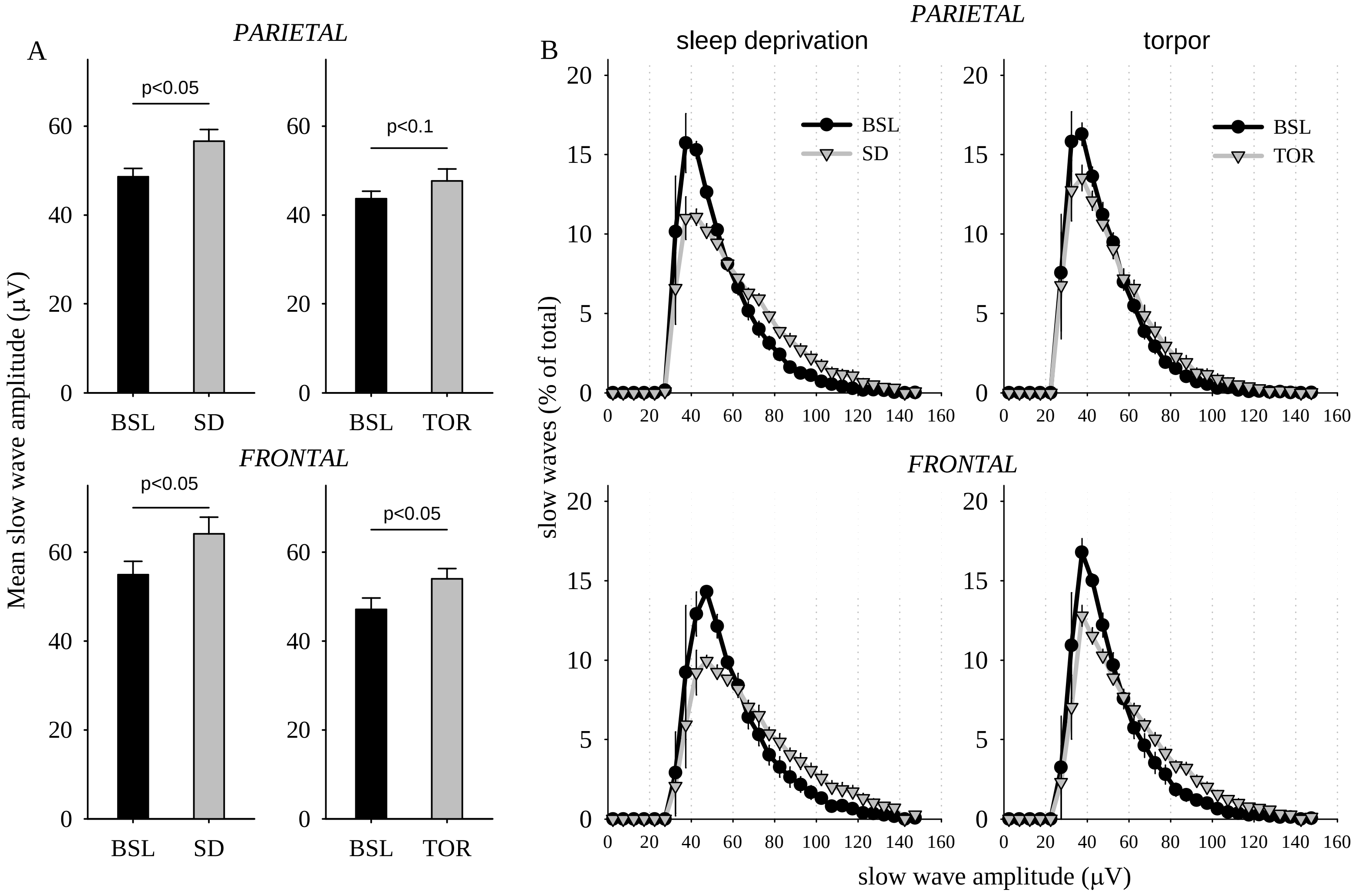

Suppl. Figure 3 (A) Effect of sleep deprivation (SD) and torpor (TOR) on the amplitude of NREM sleep slow waves. Mean values (SEM, n=6) of the amplitude of slow waves above the predefined amplitude threshold (median amplitude) occurring during the first 1-h interval after SD or TOR. P-values above the curves correspond to Wilcoxon signed-rank test (ANOVA for repeated measures: parietal, 'day' $F(1,5)=34.1$, $p=0.02$, 'condition' $F(1,5)=6.7$, $p<0.05$, 'day*condition' $F(1,5)=6.3$, $p=0.053$; frontal: 'day' $F(1,5)=50.7$, $p<0.01$, 'condition': $F(1,5)=7.9$, $p=0.04$; 'day*condition' $F(1,5)=8.2$, n.s.). (B) Distribution of NREM sleep slow waves during the first 1-h interval after SD (left panels) and after TOR (right panels), and the corresponding baseline interval, as a function of amplitude. Mean values (SEM, n=6) are shown for the parietal (top panels) and the frontal derivation (bottom panels). Note that the low amplitude slow waves (<median amplitude) are not included in these analyses.

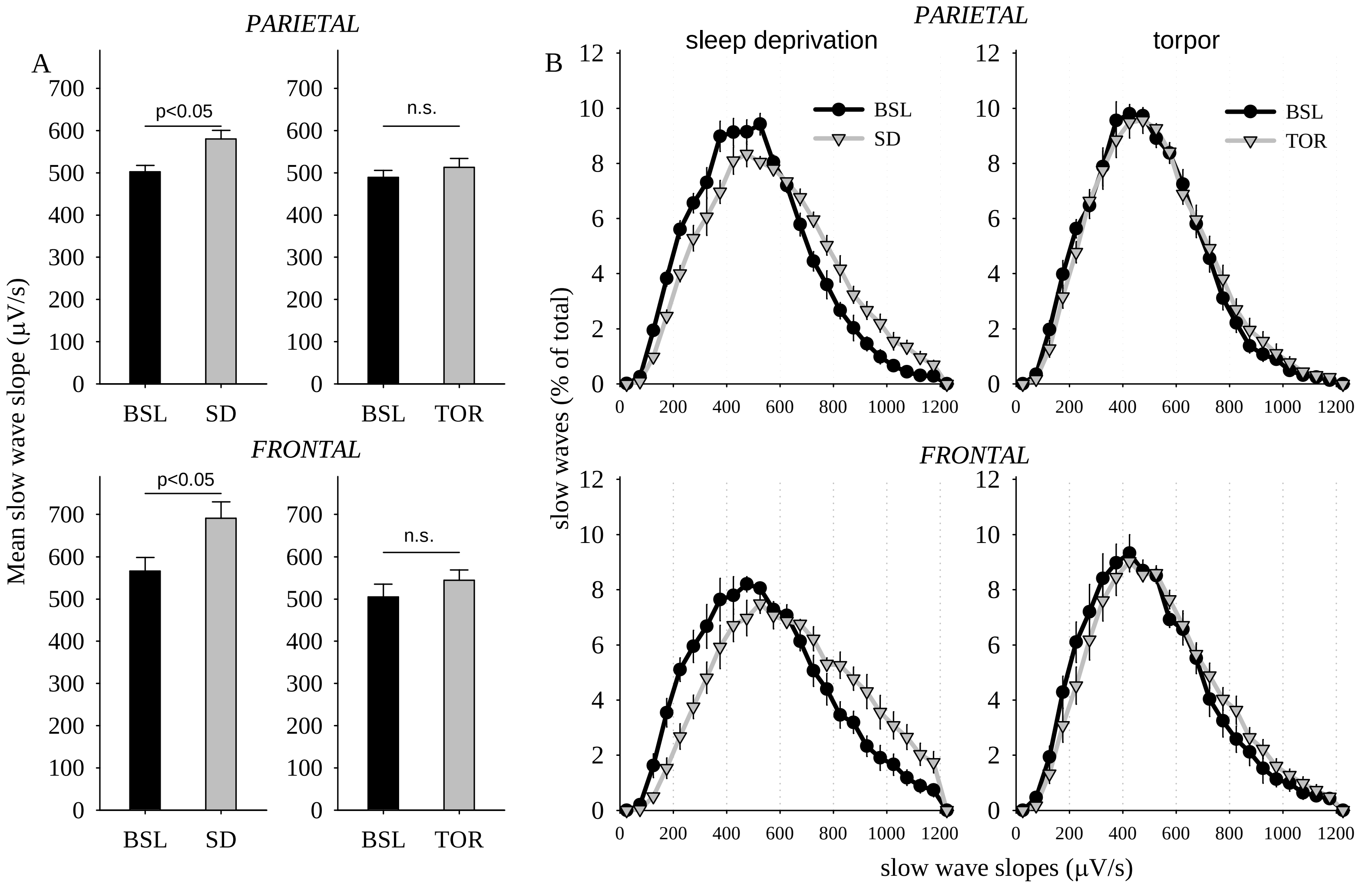

Suppl. Figure 4 (A) Effect of sleep deprivation (SD) and torpor (TOR) on the 1st slope of NREM sleep slow waves. Mean values (SEM, n=6) of the slope calculated slow waves > median amplitude occurring during the first 1-h interval after SD or TOR. P-values above the curves correspond to Wilcoxon signed-rank test (repeated measures ANOVA, parietal: 'day' $F(1,5)=32.0$, $p=0.002$, 'condition' $F(1,5)=4.2$, $p<0.1$, 'day*condition' $F(1,5)=8.7$, $p=0.03$; frontal: 'day' $F(1,5)=30.4$, $p=0.003$, 'condition' $F(1,5)=11.5$, $p=0.02$, 'day*condition' $F(1,5)=14.0$, $p=0.01$). (B) Distribution of NREM sleep slow waves during the first 1-h interval after SD (left panels) and after TOR (right panels), and the corresponding baseline interval, as a function of slope. Mean values (SEM, n=6) are shown for the parietal (top panels) and the frontal derivation (bottom panels).

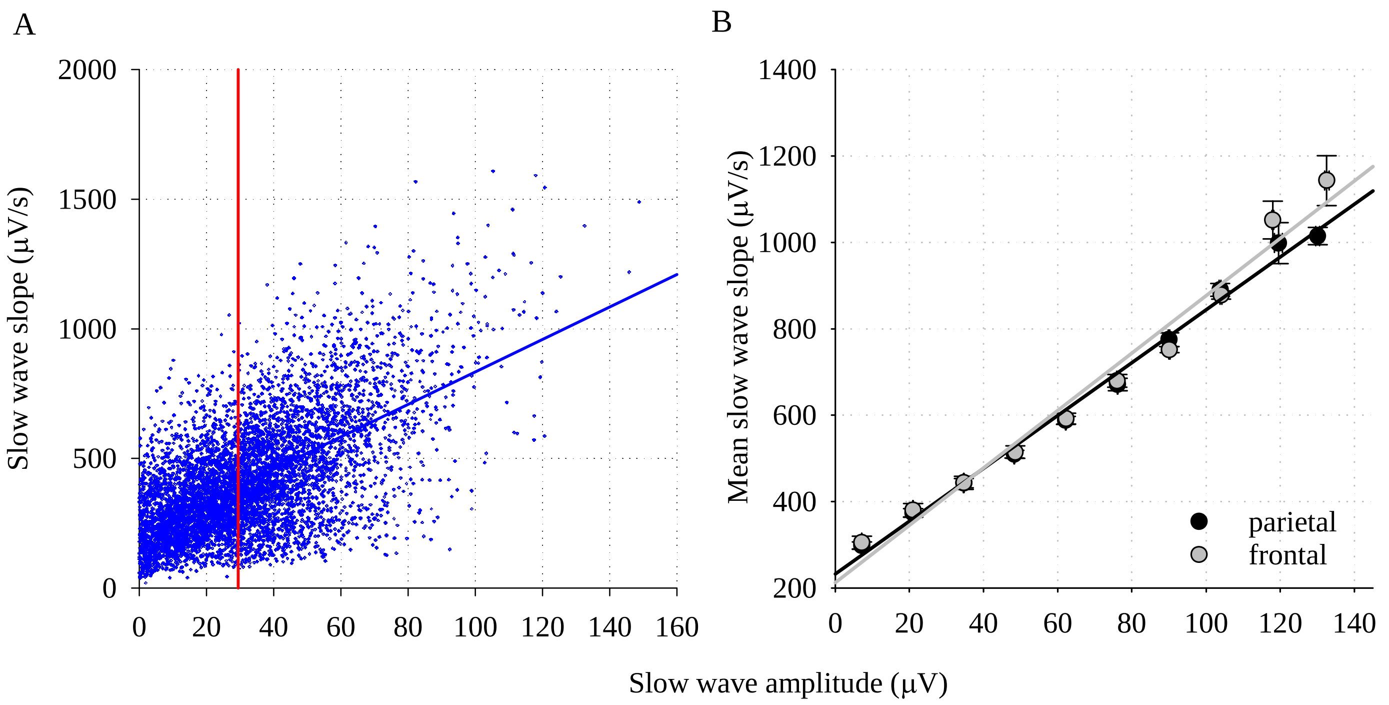

Suppl. Figure 5 (A) The relationship between slow-wave slopes and amplitude in one individual animal. All slow waves in artifact-free NREM sleep during the 1-h interval of baseline light period included in this analysis. The 1st slope of individual slopes is plotted as a function of corresponding slow-wave amplitude. Each dot corresponds to an individual slow wave. Straight blue line depicts linear regression estimated based on least squares methods. Vertical red line depicts the median amplitude value. Pearson's product moment correlation: $r=0.61$, $p<0.0001$. (B) The relationship between slow-wave amplitude and slopes. For this analysis all NREM sleep slow waves occurring during the 1-h baseline interval in each individual animal were subdivided into 10 deciles of progressively increasing amplitudes and corresponding slopes were averaged for each decile prior to calculating the mean and SEM across animals (n=6). Pearson's product moment correlation: $r=0.99$, $p<0.001$ for both derivations.

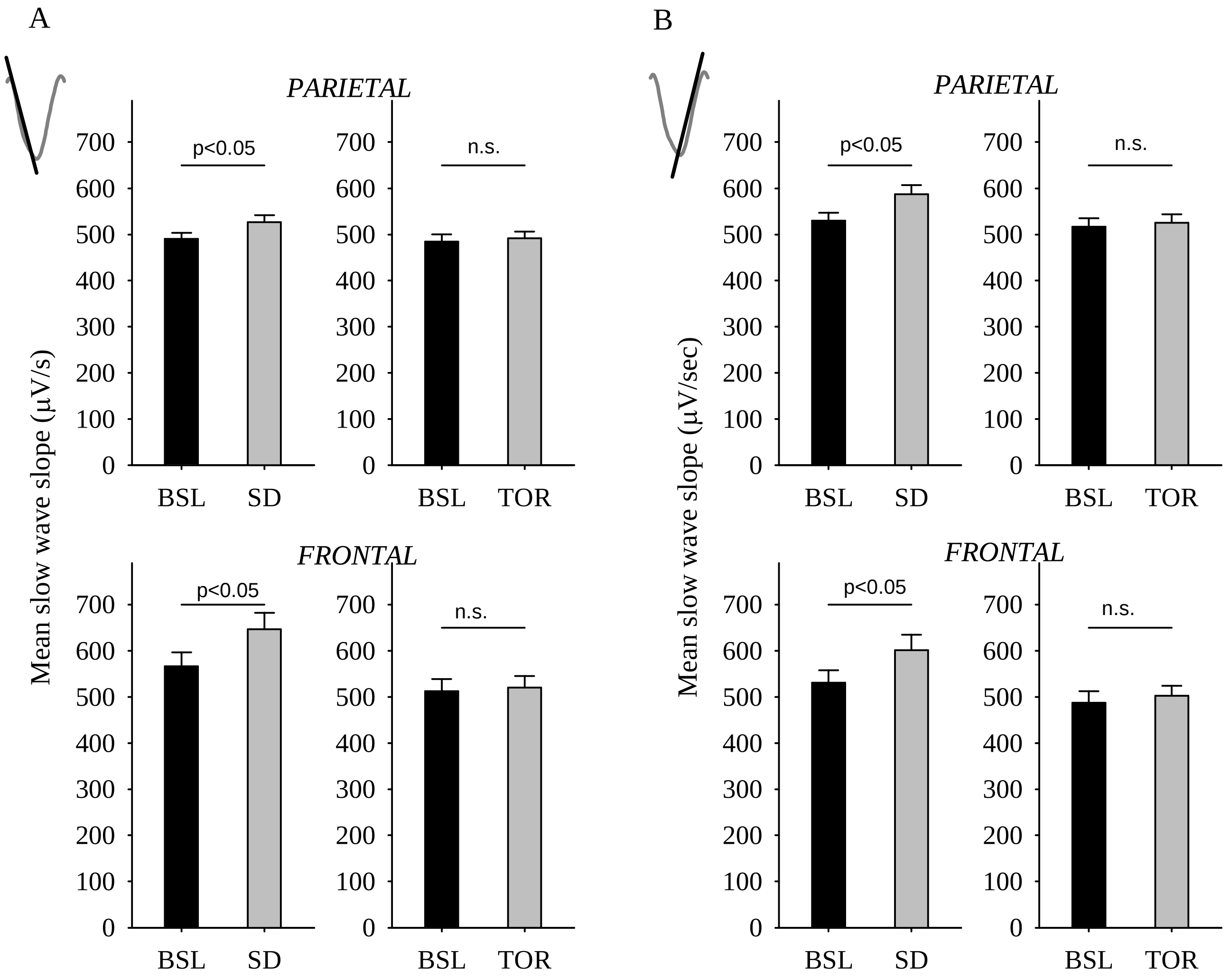

Suppl. Figure 6 (A) Effect of sleep deprivation (SD) and torpor (TOR) on the slopes of NREM sleep slow waves (slow waves > median amplitude are included, and an amplitude matching procedure was employed, see Methods section). Mean values (SEM, n=6) of the 1st slopes of slow waves occurring during the first 1-h interval after SD or TOR. P-values above the curves correspond to Wilcoxon signed-rank test. Mean values (SEM, n=6). (B) same as (A) for the 2nd slope. *1st slope*: 'condition', F(1,5)=6.1, p=0.055; 'day', F(1,5)=19.032, p=0.007, 'condition*day': F(1,5)=13.1, p=0.015, 'condition*der': F(1,5)=16.9, p=0.009; *2nd slope*: 'condition', F(1,5)=6.02, p=0.058; 'day': F(1,5)=41.76, p=0.001, 'condition*day': F(1,5)=11.3, p=0.02, 'condition*der': F(1,5)=7.9, p=0.038, 'day*der': F(1,5)=1.9, p=0.2 (n.s.).

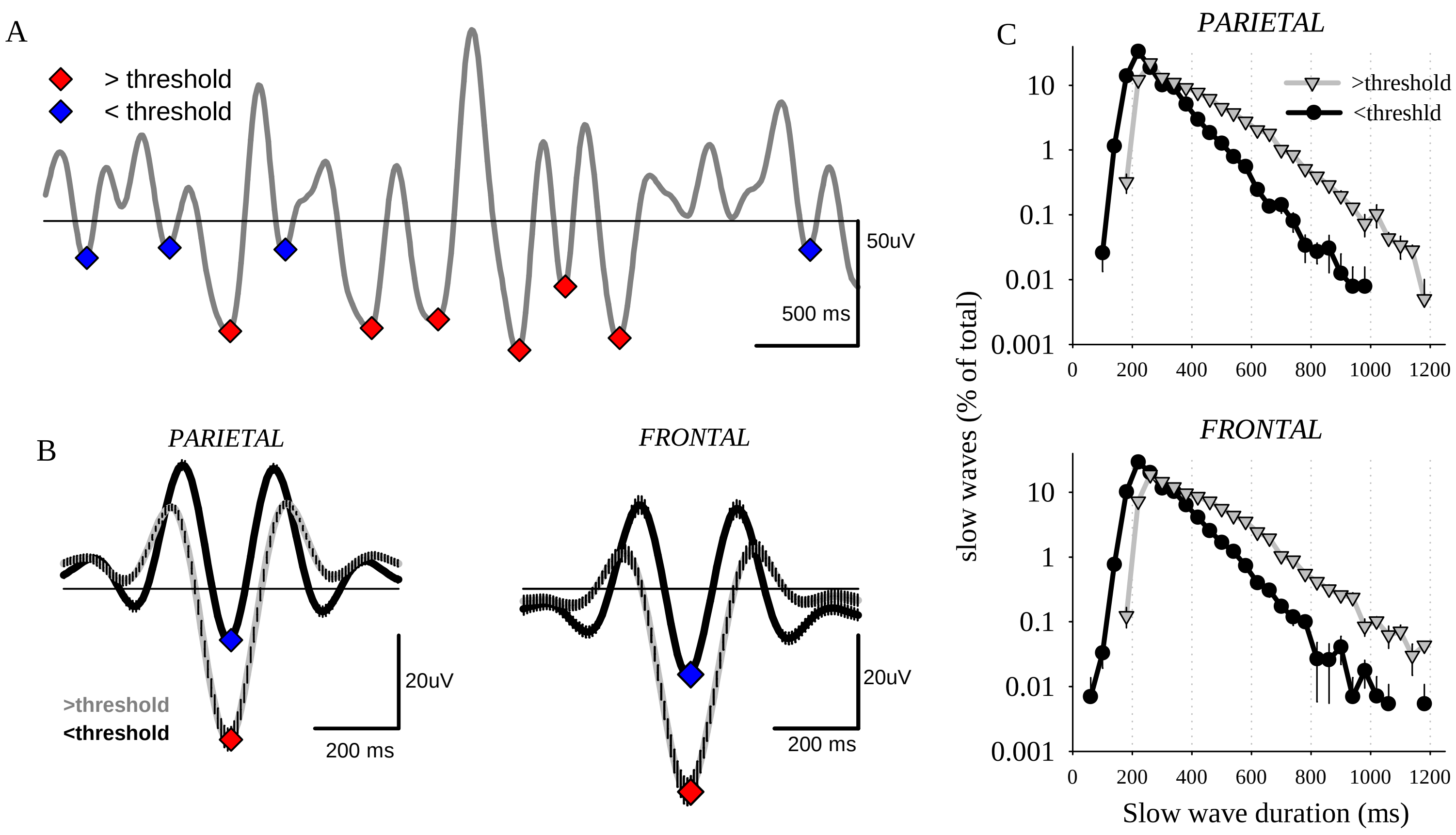

Suppl. Figure 7 (A) Representative 4-s record of the EEG (parietal derivation, filtered between 0.5-4 Hz) in NREM sleep during the baseline light period in one individual hamster. The most negative peaks of individual detected slow waves are denoted with colored symbols (red: slow waves > median amplitude, blue: slow waves < median amplitude). (B) Corresponding average waveforms of slow waves < median amplitude and > median amplitude calculated over all slow waves during the 12-h baseline period for the parietal (left) and frontal (right derivation). Mean values, SEM, n=6. (C) Distribution of slow waves as a function of their duration. The curves represent mean values (SEM, n=6) over slow waves < median amplitude and > median amplitude.
